# Agreement between attended home and ambulatory blood pressure measurements in adolescents with chronic kidney disease

**DOI:** 10.1007/s00467-022-05479-4

**Published:** 2022-02-15

**Authors:** Trevor W. Glenn, Cyd K. Eaton, Kevin J. Psoter, Michelle N. Eakin, Cozumel S. Pruette, Kristin A. Riekert, Tammy M. Brady

**Affiliations:** 1 Johns Hopkins University School of Medicine, Baltimore MD – 733 N Broadway, Baltimore, MD, 21205, USA

**Keywords:** Ambulatory blood pressure monitoring, Home blood pressure monitoring, Chronic kidney disease, Hypertension

## Abstract

**Background:**

This study aimed to compare attended home blood pressure (BP) measurements (HBPM) with ambulatory BP monitor (ABPM) readings and examine if level of agreement between measurement modalities differs overall and by subgroup.

**Methods:**

This was a secondary analysis of data from a 2-year, multicenter observational study of children 11–19 years (mean 15, SD=2.7) with chronic kidney disease. Participants had 3 standardized resting oscillometric home BPs taken by staff followed by 24-hour ABPM within 2 weeks of home BP. BP indices (measured BP/95%ile BP) were calculated for mean triplicate attended HBPM and mean ABPM measurements. Paired HBPM and ABPM measurements taken during any of 5 study visits were compared using linear regression with robust standard errors. Generalized estimating equation-based logistic regression determined sensitivity, specificity, negative, and positive predictive values with ABPM as the gold standard. Analyses were conducted for the group overall and by subgroup.

**Results:**

103 participants contributed 251 paired measurements. Indexed systolic BP did not differ between HBPM and daytime APBM (mean difference −0.002; 95% CI: −0.006, 0.003); the difference in indexed diastolic BP was minimal (mean difference −0.033; 95% CI: −0.040, −0.025). Overall agreement between HBPM and 24-hour ABPM in identifying abnormal BP was high (81.8%). HBPM had higher sensitivity (87.5%) than specificity (77.4%) and greater negative (89.8%) than positive (73.3%) predictive value, and findings were consistent in subgroups.

**Conclusions:**

Attended HBPM may be reasonable for monitoring BP when ABPM is unavailable. The greater accessibility and feasibility of attended HBPM may potentially help improve BP control among at-risk youth.

## Graphical Abstract

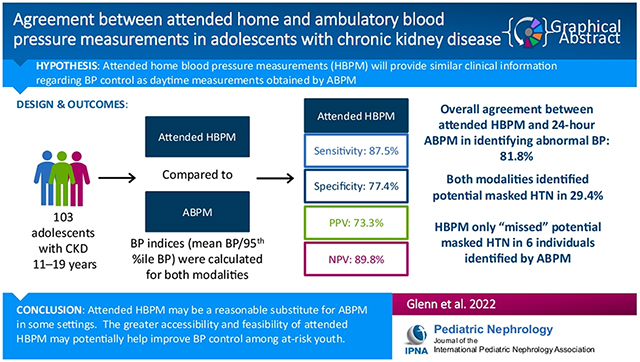

## Introduction

Hypertension, defined as a sustained elevation in blood pressure (BP) when measured repeatedly across several visits, is associated with increased cardiovascular disease (CVD) risk in both children and adults. Accurate measurement of BP and maintaining BP control is important in reducing morbidity and mortality in general, but this is especially true in children with chronic kidney disease (CKD), as hypertension is associated with CKD progression [1–5], and CVD is a leading cause of death among this population [6]. Updated pediatric guidelines recommend 24-hour ambulatory blood pressure monitoring (ABPM) to evaluate children with CKD for masked hypertension and to allow for optimized treatment of hypertension to slow progression of kidney disease [7], but this monitoring can be logistically challenging [8, 9]. ABPM devices are expensive (~$2,000 each), providers may require additional training to properly interpret results, and patients may find wearing the device for 24-hour testing and having to physically return the device to the provider’s office to be unduly burdensome. Additionally, poor tolerability of ABPM in adolescents can result in ABPM measurements that incorrectly classify an individual as hypertensive [10]. Alternative out-of-office BP readings, such as attended measurements by a pharmacist, school nurse, or community health worker, are an intriguing option for when ABPM is not feasible; however, the accuracy of using such measurements for clinical management is understudied.

The literature on the accuracy of home BP measurements (HBPM) compared to the gold-standard of ABPM has yielded varied results (with sensitivities ranging from 36–100% and specificities ranging from 44–96%) [11–22], and only two of these studies included pediatric participants [15, 22]. Attended out-of-office measurements, however, may be more standardized than the more casual home measurements and, thus, may better approximate ABPM readings. Furthermore, attended measurements may be more feasible than ABPM in low-resource settings. Thus, it is important to specifically evaluate the utility of attended BP readings (particularly in the less-studied pediatric population) as these measurements may be more practical than ABPM and more reliable than casual home measurements.

The aims of this study were as follows: 1. Evaluate how attended out-of-office HBPM measurements compare to measurements obtained by daytime ABPM (given that attended HBPM were also done during the day) in children with CKD who were prescribed an antihypertensive medication, and 2. Determine if the average of triplicate attended HBPM classifies children with abnormal BP appropriately when compared to the reference standard of 24-hour ABPM. Due to the potential influence of other characteristics known to impact BP in children, we additionally evaluated whether the associations between attended HBPM and ABPM remain when stratifying the sample by sex, overweight/obesity status, presence of a hypertension diagnosis, and/or kidney failure status [23].

## Methods

### Participants

Participants were recruited from pediatric nephrology clinics at three separate academic medical centers (Johns Hopkins Children’s Center, University of Maryland Medical Center, Children’s National Medical Center) as part of a larger 2-year observational study on medication adherence in children with CKD [24]. Each site’s Institutional Review Board approved study procedures prior to enrolling participants. Inclusion criteria included being 11–19 years old at time of consent/assent, having a physician-confirmed CKD diagnosis, and being prescribed an antihypertensive medication for >6 months (for treatment of hypertension and/or proteinuria). Participants were excluded if they were unable to comprehend English, had a sibling already participating in the study, or had cognitive impairment that would impede completion of study procedures. All participants and caregivers provided written informed assent/consent.

### Procedures

As part of the larger study, research assistants conducted study visits at participants’ homes (home visits) at baseline and every 6 months after for 2 years (total of 5 visits). These home visits took place on a day and time selected by the participant and their family, which included morning, afternoon, or evening visits on weekdays or weekends. At baseline, participants or caregivers (for participants <18 years old) reported on family demographic information. At each home visit, participant height and weight were measured along with BP using an oscillometric device, and an ABPM was initiated (see Attended Blood Pressure Measurements and Ambulatory Blood Pressure Monitoring sections for details).

Participant medical information was abstracted from their electronic medical record, including current antihypertensive medication regimens, hypertension or kidney failure diagnoses, transplant status, and serum creatinine at each provider visit proximal to the home visit. Antihypertensive medication classes prescribed to participants in the study included angiotensin-converting enzyme inhibitors, angiotensin II receptor blockers, dihydropyridine and non-dihydropyridine calcium channel blockers, beta blockers, loop and thiazide diuretics, and alpha 2 agonists. Each participant had their body mass index (BMI) calculated (kg/m^2^) from home measurements and were categorized as having overweight/obesity if their BMI was ≥ the age-sex specific 85^th^ percentile. Their estimated glomerular filtration rate (eGFR) was calculated using the CKiD bedside equation [25] for participants <18 years old and the CKD-EPI equation [26] for those ≥18 years old. Participants received monetary compensation for their time ranging from $50–100 depending on the timepoint in the larger study.

### Measures

#### Attended Blood Pressure Measurements

Attended HBPM were obtained at each home visit with a digital oscillometric BP device (OMRON Healthcare, Vernon Hills, IL, USA, IntelliSense Professional model HEM-907XL) according to standard preparation and protocol (empty bladder, no exercise/nicotine/caffeine 30 minutes prior to measurements, participant seated with back and arm supported, feet flat on the ground) [7]. Research assistants measured the participant’s mid-upper arm circumference to determine cuff size. After a 5-minute rest period, the device was programmed to measure three BP readings 30 seconds apart. Given the intrinsic variability of BP, all three measurements were averaged to provide the most reasonable estimate of resting BP.

#### Ambulatory Blood Pressure Monitoring

The ABPM device (SpaceLabs Healthcare, Snoqualmie, WA, USA, model number 90217A-1) measured participants’ BPs every 20 minutes between 8 AM–10 PM and every 30 minutes between 10 PM–8 AM. Participants were instructed to wear the ABPM device for a 24-hour period within 2 weeks following the home visit (participants chose any 24-hour period convenient for them). For the current study, average ABPM readings from participants with complete studies per current guideline [27] were analyzed for comparison with the attended BP readings.

#### Blood Pressure Index and Definition of Abnormal Blood Pressure

As BP norms vary by age, sex and height in children, and resting BP norms are typically lower than ambulatory norms, a systolic and diastolic blood pressure index (BPi) was calculated to standardize comparisons between BP measurement modalities across all children. The systolic and diastolic BPi for both attended HBPM and ABPM were calculated by taking the mean BP value and dividing by the corresponding 95^th^ percentile based on the participant’s sex, age, and height for HBPM (based on clinical practice guidelines norms [7]) and sex and height for ABPM measurements (based on established norms for ABPM [15]). A BPi ≥1 indicated an abnormal BP for HBPM. A BPi ≥1 and/or a BP load ≥25% (≥25% of BP readings >95 percentile) indicated an abnormal BP for ABPM.

### Analytic Plan

Demographic and clinical characteristics of study participants were summarized using means and standard deviations or frequencies and percentages. The analytic sample included all paired (within two weeks) attended HBPM and ABPM measurements taken during any of the 5 parent study visits; therefore, individuals could contribute more than one paired measurement. Systolic blood pressure (SBP) and diastolic blood pressure (DBP) measurements for the attended HBPM and daytime ABPM (as defined on an individual basis by participant sleep-wake journals) were summarized and compared using linear regression with robust standard errors to account for the repeated measurements of each modality for participants. Agreement between SBP and DBP for the two modalities was evaluated using Bland–Altman procedures for repeated measures and reported as the mean difference, a measure of bias, with 95% limits of agreement (LOA). Finally, classification of abnormal BP (vs. normal BP) for both SBP and DBP in children using the attended HBPM compared to the gold-standard of 24-hour ABPM was reported as the overall agreement, while diagnostic accuracy was expressed as the sensitivity, specificity, positive predictive value (PPV), and negative predictive value (NPV). Generalized estimating equations-based logistic regression analyses utilizing a working exchangeable correlation were used to estimate agreement and accuracy measures and corresponding 95% robust confidence intervals (CI). We repeated the above-described procedures for subgroups of individuals defined by sex, BMI status (overweight/obese vs. normal BMI), presence of a hypertension diagnosis (prescribed an antihypertensive for elevated blood pressure vs. isolated proteinuria), and kidney failure status (participants who had undergone kidney transplant or were on dialysis). Analyses were also conducted comparing overall agreement and performance metrics of attended HBPM to daytime ABPM measures given that HBPM readings were only done during the day. A P value <0.05 was considered statistically significant. All analyses were conducted using either STATA Version 15.1 (StataCorp, College Station, TX) or the R statistical software version 4.0.2 (R foundation for Statistical Computing, Vienna, Austria).

## Results

### Participant Characteristics

One hundred three participants from the larger CKD parent study had at least one study visit with paired ABPM and attended HBPM and were thus included in this study. Two observations from the larger study were excluded due to missing HBPM measurements, and 118 observations were excluded due to missing ABPM measurements. On average, participants were 15 years of age at the baseline visit, with 1/3 of the population female and 42% self-reporting Black race. On average, each participant contributed 2.4 visits with paired measurements resulting in a final analytic sample that consisted of 251 visits. The mean number of days between HBPM and ABPM was 4.4 (SD=4.9 days). See Table 1 for demographic characteristics of the study sample at baseline.

### Difference between the Daytime ABPM and Attended HBPM Readings

**Fig. 1:**
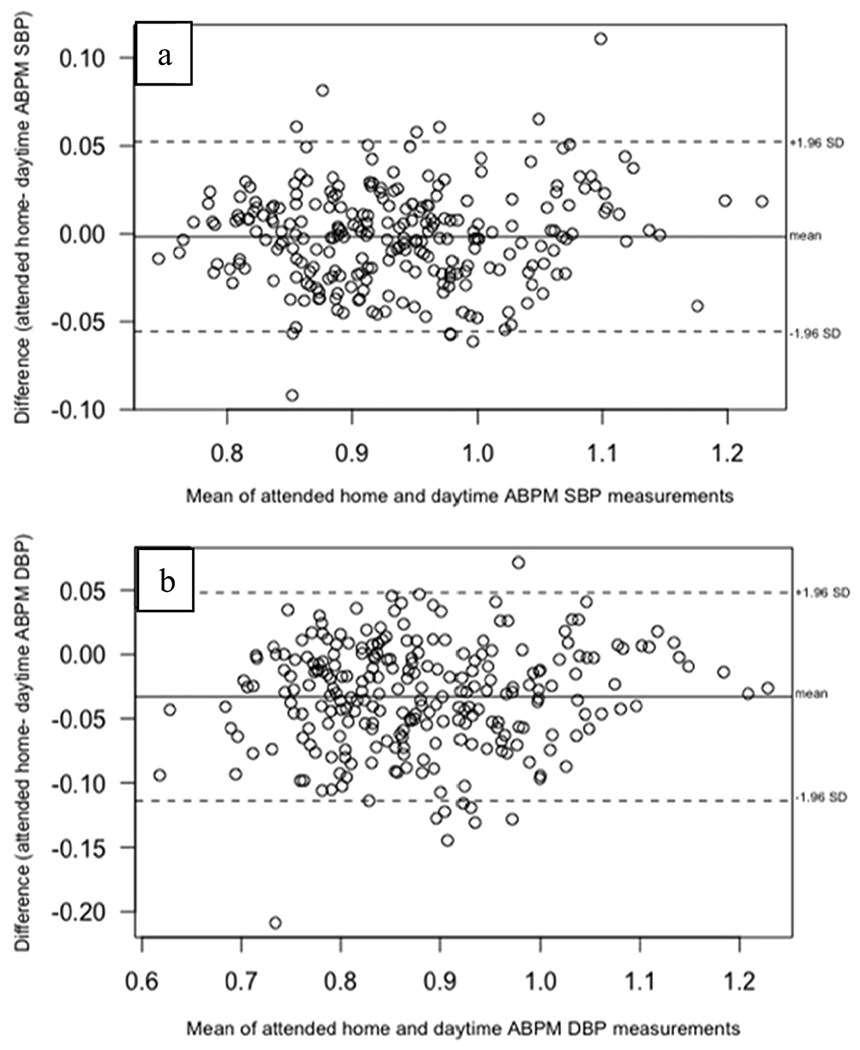
Bland–Altman plots of mean indexed SBP (panel a) and DBP (panel b) differences between attended HBPM and daytime ABPM

There was no statistically significant difference between indexed SBP measurements for daytime ABPM and attended HBPM readings. As shown in Table 2, the mean difference in SBPi between both methods was −0.002 (95% CI: −0.006, 0.003). There was a small, statistically significant difference between indexed DBP readings overall (mean difference −0.033; 95% CI: −0.040, −0.025). Figure 1 presents the Bland–Altman plots for mean indexed SBP and DBP differences between daytime ABPM and attended HBPM measurements for all participants. The LOA for SBP was (−0.056, 0.052) and the LOA for DBP was (−0.114, 0.048) and there were no indications of proportional bias across mean values of measures. The findings of minimal, non-significant differences between SBPi and small, statistically significant DBPi differences (the largest indexed DBP mean difference was −0.042 for the male subgroup) were consistent for each subgroup. We also evaluated the within-person variability of the measurements across time points in the study for each measurement modality and found that variability was minimal and similar for both ABPM and HBPM readings (data not shown).

### Identification of Abnormal BP by 24-Hour ABPM and Attended HBPM Readings

Table 3 presents the prevalence of abnormal BP as determined by 24-hour ABPM, the overall agreement between the two modalities, and the performance measures of attended HBPM as compared to the gold-standard of ABPM. 24-hour ABPM detected an abnormal BP in 41.8% of the overall paired measurements. The overall percent agreement for identifying abnormal BP between 24-hour ABPM and HBPM was high at 81.8%, with subgroup agreement ranging from 74.6% (kidney failure) to 86.4% (females). Both attended HBPM and 24-hour ABPM identified a fair number of individuals with masked hypertension: among those without a diagnosis of hypertension (who had been prescribed an antihypertensive for proteinuria), 37 (29.4%) observations had both abnormal HBPM and 24-hour ABPM. Only 6 observations in a participant without a diagnosis of hypertension were identified as having an abnormal BP by 24-hour ABPM but not by HBPM, and 16 observations had an abnormal BP by HBPM but not by 24-hour ABPM. The overall sensitivity of HBPM was high at 87.5% (and ranged from 78.9% in the overweight/obese subgroup to 93.3% in the kidney failure subgroup). The specificity was slightly lower at 77.4% (range of 48.4% in the kidney failure subgroup to 89.0% in females). NPV (89.8%) was higher than PPV (73.3%). In the subgroups, the NPV ranged from 84.4% (kidney failure) to 91.8% (in both the not overweight/obese and no hypertension diagnosis subgroups), and the PPV ranged from 67.4% (males) to 85.6% (females). Additionally, we also conducted these analyses comparing attended HBPM (which were obtained during the day) to daytime BPs from the ABPM to explore agreement without the potential confounding effect of circadian influences on BP. Those results led to attended HBPM having an even higher agreement, sensitivity, specificity, PPV, and NPV (see Supplemental Table 1).

## Discussion

In this secondary analysis of data collected from adolescents with CKD who were enrolled in a study to evaluate antihypertensive medication adherence, we found that attended BP measurements obtained in the home are comparable to measurements obtained by ABPM. There was no statistically significant difference in indexed SBP between the modalities, and the statistically significant difference in indexed DBP was minimal (mean difference of −0.033 for the overall group) and not clinically significant. The overall agreement between HBPM and 24-hour ABPM in identifying abnormal BP was high and both modalities were effective in identifying children with masked hypertension. Interestingly, ~12% of observations in youth without a previous hypertension diagnosis and a normal 24-hour ABPM could be classified as having masked hypertension based on their abnormal attended HBPM. Further, HBPM only missed 6 observations with masked hypertension identified by 24-hour ABPM measurements. These findings indicate that a normotensive HBPM result is likely to be reliable in ruling out masked hypertension whereas an abnormal HBPM result warrants further follow up. These associations were consistent among subgroups based on sex, overweight/obese status, presence of a hypertension diagnosis, and kidney failure status. Additionally, when we conducted additional analyses comparing attended HBPM with strictly daytime APBM, the overall agreement and performance metrics were even better. Taken together, attended HBPM may be helpful in monitoring BP outside the clinic setting in children with CKD when ABPM is unavailable.

There is a great need for more feasible, while still accurate, methods for monitoring out-of-office BP in the pediatric population, especially in those with CKD. While children with high BP are more likely to have hypertension and metabolic syndrome as adults [27–32], hypertension poses even greater risks for children with CKD. Children with CKD are at higher risk for the development of arrhythmias, valvular heart disease, cardiomyopathy, left ventricular hypertrophy, and vascular injury [33–35]. Additionally, cardiovascular mortality rates in young adults who developed CKD during childhood are 1000 times greater than healthy individuals, and their life expectancies are shortened by as much as 40 to 60 years [35, 36]. Therefore, it is important to be able to reliably, accurately, and regularly monitor BP in pediatric populations to optimize treatment, which will serve to delay the progression of CKD and minimize the associated increased cardiovascular morbidity and mortality. Our findings suggest that attended HBPM may be a useful out-of-office measurement modality to promote better long-term health for those at risk for complications associated with high BP.

While prior studies and literature reviews have compared other methods for monitoring BP (self-measured automatic at home, unattended automatic in clinic, manual in clinic) with ABPM [37–39], results have varied greatly, and many studies have focused on adult rather than pediatric populations. Some studies found that self-measured home rather than clinic BP measurements more closely reflected ABPM readings [11, 15, 22, 40]. It has not been well studied, however, how attended out-of-office BP readings (rather than unattended, self- or parent-measured home readings) compare to ABPM. Our study was able to demonstrate good agreement between ABPM and attended HBPM in the identification of abnormal BP in a pediatric sample with CKD. Additionally, our study explored whether subgroups of youth with CKD – youth at greater CVD risk due to their childhood BP trajectories or greater BP variability – had different agreement between the two BP measuring modalities [24, 40, 41]. We found little difference in results when stratifying children into groups by characteristics known to impact BP in children when compared to the results for the overall sample. The group with kidney failure, however, did have a lower overall agreement and specificity compared to other groups, which indicates that attended HBPM may not be as comparable to ABPM in that population.

While many methods exist to monitor BP in children and adolescents, ABPM continues to be considered the gold-standard in pediatrics [7]. However, that modality can be logistically difficult to implement on a broad scale and is poorly tolerated by some, which can impact the measurements obtained [8, 10]. Attended BP measurements by trained individuals offer an advantage over the often-used unattended self- or parent-obtained home BP measurements, as attended measurements can optimize standardization with proper patient preparation (empty bladder, no exercise/nicotine/caffeine for 30 minutes prior, rest for 5 minutes before initiating measurements, individualized cuff selection) and proper patient positioning (back/feet/arm supported with cuff on upper arm positioned over brachial artery). While attended BP measurements are logistically more involved than self-measured home readings, attended out-of-office BP measurements may be particularly helpful for those who are unable to afford a home BP monitor or do not have access to one, for children who are unable or unwilling to come to the clinic frequently for BP checks, and for those who are unable or unwilling to do ABPM due to intolerance or distance to clinic. Attended out-of-office BP measurements may also be less resource intensive than ABPM. Some families may find it easier to access a school nurse’s office or pharmacy for attended measurements from a trained individual than to present to a doctor’s office during set hours to pick up an ABPM, receive education on device operation, await device initialization, and then complete measurements and diary information at home, followed by re-presenting to the office for device return and results interpretation. The challenge to obtaining complete, reliable ABPM from adolescents is underscored by the large number of HBPM measures that did not have a corresponding ABPM in this study, despite the provision of home delivery/pick up of devices by research staff. Additionally, there are a wide variety of healthcare workers in numerous community settings who could potentially oversee attended BP measurements, such as school nurses, community health workers, or pharmacists, making attended out-of-office BP measurements a potential low-cost and feasible adjuvant to the monitoring of BP accurately and regularly in children with CKD.

Limitations to this study include an absence of clinic BP, manual BP readings, and unattended, self- or parent-measured home BP for additional comparisons, and the duration of up to two weeks between attended HBPM and ABPM measurements (although the mean number of days between readings was 4.3). Additionally, although participants were recruited from three separate sites, the sites were in the same geographical region, and all participants were children 11–19 years of age with CKD, both of which may potentially limit generalizability. While the attended HBPM were taken out-of-office, there may be an element of white coat hypertension given the supervised nature of the measurements, and white coat hypertension (and its potential influence on the study’s results) is not well understood outside of the clinic setting or as it relates to non-physician attended readings. Despite these limitations, this study has many strengths, including a diverse sample, repeated timepoints over 2 years, and indexed BPs for better comparison between the two modalities. Furthermore, our study examined attended out-of-office BP measurements, which have not been thoroughly evaluated in the existing literature. Also, even though the out-of-office BP measurements were taken in triplicate on one day rather than having repeated measures over multiples days and nights (as could be more easily done with unattended HBPM readings), they showed good agreement with ABPM.

## Conclusion

In summary, attended out-of-office BP measurements, which could be done by a school nurse, pharmacist, or other community health worker, may be helpful in monitoring BP in children with CKD or other high-risk conditions. Given the higher NPV than PPV, attended HBPM readings that are normotensive may more reliably indicate good BP control while abnormal readings may warrant further follow up. Attended out-of-office BP measurements may be logistically easier than ABPM and more feasible to do in low-resource settings with limited or no access to home BP monitors, all the while maintaining accurate BP readings.

## Supplementary Material

1788172_Sup_s1

## Figures and Tables

**Table 1.** Demographic and clinical characteristics of study sample

	Overall (N=103)
Age (years), mean (SD)	15.0 (2.69)
Sex, n (%)	
Male	69 (67)
Female	34 (33)
Race/Ethnicity, n (%)	
White	38 (37)
Black	43 (42)
Asian	2 (2)
Latinx	2 (2)
Other	18 (18)
Overweight/Obese at baseline, n (%)	
No	63 (61)
Yes	40 (39)
Body mass index, mean in kg/m^2^ (SD)	23.9 (6.74)
Hypertension diagnosis, n (%)	
No	53 (52)
Yes	50 (48)
Glomerular diagnosis, n (%)	
No	56 (54)
Yes	47 (46)
Kidney failure, n (%)	
No	72 (70)
Yes	31 (30)
Kidney transplant, n (%)	
No	78 (76)
Yes	25 (24)
First recorded eGFR, mean (SD)^a^	64.8 (34.5)

eGFR = estimated glomerular filtration rate in ml/min/1.73 m^2^ based on the bedside CKiD equation for those <18 years and the CKD-EPI equation for those ≥18 years old. SD=standard deviation.

a n=79

**Table 2.** Differences and limits of agreement between the mean indexed blood pressures (attended HBPM vs. daytime ABPM) among children with chronic kidney disease, overall and by subgroups

		Attended HBPM	Daytime ABPM	Mean difference	P value	Limits of agreement
**Overall (n=103; 251 visits)**	**Systolic, mean (SD)**	0.94 (0.09)	0.94 (0.09)	−0.002	0.49	(−0.056, 0.052)
	**Diastolic, mean (SD)**	0.86 (0.11)	0.90 (0.11)	−0.033	<0.001	(−0.114, 0.048)
						
**Males (n=69; 162 visits)**	**Systolic, mean (SD)**	0.93 (0.10)	0.93 (0.09)	−0.001	0.67	(−0.060, 0.057)
	**Diastolic, mean (SD)**	0.85 (0.12)	0.89 (0.11)	−0.042	<0.001	(−0.129, 0.045)
**Females (n=34; 89 visits)**	**Systolic, mean (SD)**	0.94 (0.09)	0.95 (0.08)	−0.002	0.53	(−0.047, 0.043)
	**Diastolic, mean (SD)**	0.88 (0.10)	0.90 (0.10)	−0.016	<0.001	(−0.072, 0.040)
						
**Overweight/Obese (n=40; 102 visits)**	**Systolic, mean (SD)**	0.93 (0.09)	0.92 (0.08)	0.001	0.86	(−0.045, 0.046)
	**Diastolic, mean (SD)**	0.85 (0.10)	0.88 (0.10)	−0.027	<0.001	(−0.106, 0.051)
**Not Overweight/Obese (n=63; 149 visits)**	**Systolic, mean (SD)**	0.94 (0.09)	0.95 (0.09)	−0.003	0.34	(−0.062, 0.056)
	**Diastolic, mean (SD)**	0.87 (0.12)	0.91 (0.11)	−0.037	<0.001	(−0.119, 0.045)
						
**Hypertension Diagnosis (n=50; 125 visits)**	**Systolic, mean (SD)**	0.96 (0.10)	0.96 (0.09)	0.001	0.86	(−0.058, 0.059)
	**Diastolic, mean (SD)**	0.89 (0.12)	0.92 (0.11)	−0.036	<0.001	(−0.123, 0.052)
**No Hypertension Diagnosis (n=53; 126 visits)**	**Systolic, mean (SD)**	0.91 (0.08)	0.92 (0.08)	−0.004	0.15	(−0.053, 0.045)
	**Diastolic, mean (SD)**	0.84 (0.10)	0.87 (0.10)	−0.030	<0.001	(−0.104, 0.043)
						
**Kidney failure (n=31; 78 visits)**	**Systolic, mean (SD)**	0.97 (0.08)	0.97 (0.09)	−0.006	0.167	(−0.064, 0.052)
	**Diastolic, mean (SD)**	0.94 (0.11)	0.90 (0.13)	−0.041	<0.001	(−0.132, 0.051)
**No kidney failure (n=72; 173 visits)**	**Systolic, mean (SD)**	0.92 (0.09)	0.92 (0.09)	0.000	0.862	(−0.052, 0.053)
	**Diastolic, mean (SD)**	0.88 (0.10)	0.85 (0.10)	−0.029	<0.001	(−0.105, 0.046)

HBPM: home blood pressure measurements; ABPM: ambulatory blood pressure monitor(ing); SD: standard deviation

BP index is calculated as the mean BP/95^th^ %ile BP; a BP index ≥1 is abnormal.

**Table 3.** Agreement and performance measures of attended HBPM compared to 24-hour ABPM among children with chronic kidney disease

	Number of observations (n, %)	Prevalence of Abnormal BP by 24-hour ABPM (95% CI)	Overall agreement between 24-hour ABPM and home BP on identification of abnonnal BP	True Positive^a^ (# of obs)	False Positive^a^ (# of obs)	True Negative^a^ (# of obs)	False Negative^a^ (# of obs)	Sensitivity of attended BP readings (95% CI)	Specificity of attended BP readings (95% CI)	PPV of attended BP readings (95% CI)	NPV of attended BP readings (95% CI)
Overall	251, 100%	41.8% (36.3, 47.3%)	81.8% (76.4, 87.1%)	36.7% (n=92)	13.6% (n=34)	44.6% (n=112)	5.2% (n=13)	87.5% (81.1, 94.0%)	77.4% (69.1, 85.7%)	73.3% (64.1, 82.5%)	89.8% (84.6, 94.9%)
	
Males	162, 64%	39.9% (33.1, 46.7%)	79.2% (72.2, 86.1%)	35.6% (n=58)	17.2% (n=28)	42.9% (n=70)	4.3% (n=7)	89.2% (81.6, 96.9%)	71.8% (61.0, 82.7%)	67.4% (56.2, 78.6%)	91.0% (84.6, 97.4%)
Females	89, 36%	45.5% (36.5, 54.4%)	86.4% (78.6, 94.3%)	38.6% (n=34)	6.8% (n=6)	47.7% (n=42)	6.8% (n=6)	84.7% (73.0, 96.4%)	89.0% (78.5, 99.6%)	85.6% (70.9, 100.0%)	87.8% (79.1, 96.5%)
	
Overweight/Obese	102, 41%	36.3% (27.3, 45.2%)	81.8% (73.4, 90.1%)	28.4% (n=29)	10.8% (n=11)	52.9% (n=54)	7.8% (n=8)	78.9% (67.6, 90.2%)	83.5% (72.0, 95.1%)	77.1% (61.9, 92.2%)	87.4% (79.4, 95.4%)
Not Overweight/Obese	149, 59%	45.6% (39.0, 52.3%)	81.2% (74.4, 88.0%)	42.3% (n=63)	15.4% (n=23)	38.9% (n=58)	3.4% (n=5)	92.5% (86.1, 99.0%)	71.7% (60.7, 82.7%)	72.6% (62.2, 83.1%)	91.8% (84.8, 98.9%)
	
Hypertensive Diagnosis	125, 50%	49.6% (41.2, 58.0%)	80.8% (73.0, 88.5%)	44.0% (n=55)	14.4% (n=18)	36.0% (n=45)	5.6% (n=7)	88.2% (79.7, 96.7%)	72.7% (59.0, 86.3%)	76.2% (64.0, 88.5%)	87.0% (78.3, 95.7%)
No Hypertensive Diagnosis	126, 50%	34.1% (27.0, 41.3%)	82.7% (75.4, 90.0%)	29.4% (n=37)	12.7% (n=16)	53.2% (n=67)	4.8% (n=6)	86.4% (76.5, 96.3%)	80.8% (70.5, 91.2%)	69.8% (55.9, 83.7%)	91.8% (85.6, 98.1%)
											
Kidney failure	78, 31%	57.7% (47.0, 68.3%)	74.6% (64.6, 84.7%)	53.9% (n=42)	21.8% (n=17)	20.5% (n=16)	3.9% (n=3)	93.3% (86.3, 100.0%)	48.4% (31.1, 65.8%)	71.8% (59.2, 84.5%)	84.4% (67.7, 100.0%)
No kidney failure	173, 69%	34.7% (28.3, 41.0%)	84.7% (78.6, 90.8%)	28.9% (n=50)	9.8% (n=17)	55.5% (n=96)	5.8% (n=10)	83.4% (73.9, 92.9%)	85.5% (77.4, 93.5%)	75.8% (63.8, 87.9%)	90.7% (85.4, 96.1%)

ABPM: ambulatory blood pressure monitor(ing); BP: blood pressure; CI: confidence interval; HBPM: home blood pressure measurements; PPV: positive predictive value; NPV: negative predictive value; # of obs: number of observations

a True positive is elevated BP on both attended HBPM and ABPM. False positive is elevated BP on attended HBPM but not on APBM. True negative is normal BP on attended HBPM and ABPM. False negative is normal on attended HBPM but not on ABPM.

Abnormal BP by ABPM is defined as a mean BP≥ age-height specific 95^th^percentile and/or a BP load ≥25%.

Overweight/obese is defined as a BMI ≥ age-sex specific 85^th^ percentile.

## Abbreviations:

ABPM: ambulatory blood pressure monitor
BMI: body mass index
BP: blood pressure
BPi: blood pressure index
CI: confidence interval
CKD: chronic kidney disease
DBP: diastolic blood pressure
eGFR: estimated glomerular filtration rate
HBPM: home blood pressure measurements
LOA: limits of agreement
NPV: negative predictive value
PPV: positive predictive value
SBP: systolic blood pressure
