# Supplementary material for: Agreement between attended home and ambulatory blood pressure measurements in adolescents with chronic kidney disease
Source: Pediatr Nephrol. Author manuscript; Available in PMC 2023 Oct 1. (PMC9376201; doi:10.1007/s00467-022-05479-4)
Supplement: 1788172_Sup_s1 [file NIHMS1788172-supplement-1788172_Sup_s1.docx]

**Supplemental Table 1**. Agreement and performance metrics of attended HBPM compared to daytime ABPM in kids with chronic kidney disease

|  | Number of observations (n, %) | Prevalence of Abnormal BP by daytime ABPM (95% CI) | Overall agreement between daytime ABPM and home BP on identification of abnormal BP | True Positive^a^ (# of obs) | False Positive^a^ (# of obs) | True Negative^a^ (# of obs) | False Negative^a^ (# of obs) | Sensitivity of attended BP readings (95% CI) | Specificity of attended BP readings (95% CI) | PPV of attended BP readings (95% CI) | NPV of attended BP readings (95% CI) |
| --- | --- | --- | --- | --- | --- | --- | --- | --- | --- | --- | --- |
| Overall | 251, 100% | 41.8% (37.2, 46.4%) | 88.5% (84.1, 92.9%) | 40%  (n=101) | 10%  (n=25) | 48%  (n=121) | 2%  (n=4) | 96.2% (92.5, 99.9%) | 82.6% (75.5, 89.8%) | 79.0% (70.4, 87.6%) | 96.8% (93.8, 99.9%) |
|  |  | | | | | | | | | | |
| Males | 162, 64% | 40.5% (34.0, 47.0%) | 86.6% (80.4, 92.8%) | 39.9% (n=65) | 12.9% (n=21) | 46.6% (n=76) | 0.6% (n=1) | 98.3% (95.1, 100.0%) | 78.0% (68.2, 87.9%) | 72.7% (60.7, 84.8%) | 98.7% (96.1, 100.0%) |
| Females | 89, 36% | 44.3% (39.1, 49.5%) | 92.0% (86.9, 97.1%) | 40.9% (n=36) | 4.6% (n=4) | 51.1% (n=49) | 3.4% (n=3) | 91.3% (81.3, 100.0%) | 92.0% (84.5, 99.5%) | 89.9% (81.1, 98.7%) | 93.8% (87.1, 100.0%) |
|  |  | | | | | | | | | | |
| Overweight/ Obese | 102, 41% | 37.3% (31.9, 42.6%) | 91.8% (86.9, 96.7%) | 34.3% (n=35) | 4.9% (n=5) | 57.8% (n=59) | 2.9% (n=3) | 90.8% (82.6, 99.0%) | 92.2% (85.4, 98.9%) | 87.6% (78.0, 97.3%) | 95.2% (89.9, 100.0%) |
| Not Overweight/ Obese | 149, 59% | 45.0% (38.3, 51.6%) | 85.5% (78.9, 92.1%) | 44.3% (n=66) | 13.4% (n=20) | 41.6% (n=62) | 0.7% (n=1) | 98.5% (95.4, 100.0%) | 76.0 (65.6, 86.3%) | 74.6% (63.5, 85.8%) | 98.4% (95.2, 100.0%) |
|  |  | | | | | | | | | | |
| Hypertensive Diagnosis | 125, 50% | 51.2% (44.5, 57.9%) | 88.0% (82.0, 94.0%) | 48.8% (n=61) | 9.6% (n=12) | 39.2% (n=49) | 2.4% (n=3) | 95.4% (90.3, 100.0%) | 79.4% (67.9, 90.9%) | 82.9% (72.7, 93.1%) | 94.3% (88.0, 100.0%) |
| No Hypertensive Diagnosis | 126, 50% | 32.5% (26.3, 38.8%) | 89.0% (82.5, 95.6%) | 31.8% (n=40) | 10.3% (n=13) | 57.1% (n=72) | 0.8% (n=1) | 97.5 (92.4, 100.0%) | 85.1% (76.0, 94.2%) | 74.0% (59.5, 88.5%) | 98.6% (96.0, 100.0%) |
|  |  |  |  |  |  |  |  |  |  |  |  |
| Kidney failure | 78, 31% | 68.7%  (58.1, 79.2%) | 83.7% (74.3, 93.1%) | 66.3%  (n=55) | 13.3%  (n=11) | 18.1%  (n=15) | 2.4%  (n=2) | 96.5% (91.8, 100.0%) | 57.9% (37.9, 77.9%) | 81.3% (68.6, 94.0%) | 88.3% (72.9, 100.0%) |
| No kidney failure | 173, 69% | 28.6% (24.0, 33.2%) | 90.5% (85.5, 95.4%) | 27.4%  (n=46) | 8.3%  (n=14) | 63.1%  (n=106) | 1.2%  (n=2) | 95.7% (89.5, 100.0%) | 87.9% (81.1, 94.7%) | 76.2% (64.1, 88.3%) | 98.1% (95.6, 100.0%) |

ABPM: ambulatory blood pressure monitor(ing); BP: blood pressure; CI: confidence interval; HBPM: home blood pressure measurements; PPV: positive predictive value; NPV: negative predictive value; # of obs: number of observations
^a^ True positive is elevated BP on both attended HBPM and ABPM. False positive is elevated BP on attended HBPM but not on APBM. True negative is normal BP on attended HBPM and ABPM. False negative is normal on attended HBPM but not on ABPM.
Abnormal BP by ABPM is defined as a mean daytime BP≥ age-height specific 95^th^ percentile and/or a BP load ≥25%.
Overweight/obese is defined as a BMI ≥ age-sex specific 85^th^ percentile.
